# Convergent Evolution and Predictability of Gene Copy Numbers Associated with Diets in Mammals

**DOI:** 10.1093/gbe/evaf008

**Published:** 2025-01-24

**Authors:** Kayla Wilhoit, Shun Yamanouchi, Bo-Jyun Chen, Yo Y Yamasaki, Asano Ishikawa, Jun Inoue, Wataru Iwasaki, Jun Kitano

**Affiliations:** Ecological Genetics Laboratory, National Institute of Genetics, Mishima, Shizuoka 411-8540, Japan; Biomedical Sciences Program, Texas A&M University, College Station, TX, USA; University Program in Genetics and Genomics, Duke University, Durham, NC, USA; Department of Biological Sciences, Graduate School of Science, The University of Tokyo, Tokyo 113-0032, Japan; Ecological Genetics Laboratory, National Institute of Genetics, Mishima, Shizuoka 411-8540, Japan; Genetics Course, The Graduate University for Advanced Studies, Mishima, Shizuoka 411-8540, Japan; Ecological Genetics Laboratory, National Institute of Genetics, Mishima, Shizuoka 411-8540, Japan; Genetics Course, The Graduate University for Advanced Studies, Mishima, Shizuoka 411-8540, Japan; Department of Integrated Biosciences, Graduate School of Frontier Sciences, The University of Tokyo, Kashiwa, Chiba 277-0882, Japan; Atmosphere and Ocean Research Institute, The University of Tokyo, Kashiwa, Chiba 277-0882, Japan; Department of Biological Sciences, Graduate School of Science, The University of Tokyo, Tokyo 113-0032, Japan; Department of Integrated Biosciences, Graduate School of Frontier Sciences, The University of Tokyo, Kashiwa, Chiba 277-0882, Japan; Atmosphere and Ocean Research Institute, The University of Tokyo, Kashiwa, Chiba 277-0882, Japan; Ecological Genetics Laboratory, National Institute of Genetics, Mishima, Shizuoka 411-8540, Japan; Genetics Course, The Graduate University for Advanced Studies, Mishima, Shizuoka 411-8540, Japan

**Keywords:** convergent evolution, phylogenetic signals, copy number variation, trophic level, trophic position, gene duplication

## Abstract

Convergent evolution, the evolution of the same or similar phenotypes in phylogenetically independent lineages, is a widespread phenomenon in nature. If the genetic basis for convergent evolution is predictable to some extent, it may be possible to infer organismic phenotypes and the capability of organisms to utilize new ecological resources based on genome sequence data. While repeated amino acid changes have been studied in association with convergent evolution, relatively little is known about the potential contribution of repeated gene copy number changes. In this study, we explore whether gene copy number changes of particular gene families are linked to diet shifts in mammals and assess whether trophic ecology can be inferred from the copy numbers of a specific set of gene families. Using 86 mammalian genome sequences, we identified 24 gene families with a trend toward higher copy numbers in herbivores, carnivores, and omnivores, even after phylogenetic corrections. We were able to confirm previous findings on genes such as amylase, olfactory receptors, and xenobiotic metabolism genes, and identify novel gene families whose copy numbers correlate with dietary patterns. For example, omnivores exhibited higher copy numbers of genes encoding regulators of translation. We also established a discriminant function based on the copy numbers of 13 gene families that can help predict trophic ecology to some extent. These findings highlight a possible association between convergent evolution and repeated copy number changes in specific gene families, suggesting the potential to develop a method for predicting animal ecology from genome sequence data.

**Significance**

The role of repeated amino acid changes in convergent evolution is well documented, but relatively little is known about the potential contribution of repeated gene copy number changes to convergent evolution. Using 86 mammalian genomes, we identified gene families with a trend toward higher gene copy numbers in herbivores, carnivores, and omnivores, revealing a potential link between specific gene families and dietary patterns. Our findings suggest the possibility of predicting mammal ecology and adaptability through analysis of the copy numbers of key gene families associated with convergent evolution.

## Introduction

Convergent evolution, the evolution of the same or similar phenotypes in phylogenetically independent lineages, is prevalent in nature (Schluter 2000; Blount et al. 2018). An increasing number of case studies have demonstrated that the same genes and even the same mutations often underlie convergent evolution (Hoekstra and Coyne 2007; Christin et al. 2010; Conte et al. 2012; Martin and Orgogozo 2013; Storz 2016; Losos 2017). If the genetic basis for convergent evolution is predictable to some extent, it may be possible to infer organismic phenotypes and the capability of an organism to utilize new ecological resources from genome sequence data. For example, when particular amino acid changes occur repeatedly in convergent evolution, organismic phenotypes can be predicted based on protein sequence information (Christin et al. 2010; Ujvari et al. 2015; Storz 2016; Fukushima and Pollock 2023; Taverner et al. 2019; Karageorgi et al. 2019). Gene copy number changes are also often associated with convergent evolution, such as the convergent evolution of drug resistance in insects (You et al. 2013; Bansal and Michel 2018; War et al. 2018) and plants (Patterson et al. 2018), dietary adaptation in mammals (Perry et al. 2007; Pajic et al. 2019), and freshwater adaptation in fish (Ishikawa et al. 2019). However, we do not know how informative the copy numbers of specific genes are for predicting the organismic phenotypes and ecology.

Diet shifts have repeatedly occurred across the animal tree of life (Román-Palacios et al. 2019; Amador and Giannini 2021). For example, mammalian species exploit diverse diets, and transitions among herbivores, omnivores, and carnivores have occurred repeatedly in mammals (Price et al. 2012). Information on mutations that repeatedly occur during diet shifts may enable us to predict whether specific organisms have the genetic predisposition to shift their diets based on genome sequence data. Such information would be applicable to livestock (Gaughan et al. 2019; Courtier-Orgogozo and Martin 2020) and could help predict the geographical distribution of organisms and the patterns of predator–prey interactions under ecological disturbances and climate changes in the future (Bellard et al. 2012; Albouy et al. 2014). Furthermore, investigation of the functions of genes whose evolutionary changes have repeatedly occurred during diet shifts can provide valuable insights into the selective pressures that constrain ecological niche shifts.

Changes in gene copy numbers can contribute to diet shifts through three possible mechanisms. First, copy number increases in particular genes or gene family expansions are often associated with the increased efficiency of utilization of energy sources rich in new diets. For example, copy number increases of amylase genes occur in populations and species that utilize starch-rich diets, including human populations in agricultural societies (Perry et al. 2007), human-affiliated domestic animals (Axelsson et al. 2013; Skoglund et al. 2015), and omnivorous mammals (Pajic et al. 2019). Because the amylase copy number expansion in humans predates the development of agriculture (Mathieson and Mathieson 2018; Yilmaz et al. 2024), selection might act on the preexisting copy number variations. Increases in the copy numbers of the amylase genes may lead to the amylase gene expression in saliva and facilitate the hydrolysis of starch and the absorbance of carbohydrates (Berg et al. 2015; Pajic et al. 2019). The amplification of type I interferon gene families in the cow may lead to the evolution of new interferon genes, which may contribute to the maintenance of fermenting bacteria through their antiviral function (Walker and Roberts 2009). A duplicated copy of a ribonuclease gene in a leaf-eating monkey enhanced a ribonucleolytic activity by amino acid changes and may contribute to the digestion of intestinal bacteria (Zhang et al. 2002).

Second, gene copy number increases or gene family expansions are often associated with the nullification of toxic compounds present in new diets. For example, plants often produce secondary compounds to deter attack by herbivores (Harborne 2014). Several herbivorous insects have amplified genes encoding enzymes, which may diversify in functions and enable animals to detoxify a wide range of plant-derived toxins (You et al. 2013; Bansal and Michel 2018; War et al. 2018). Reduced copy numbers of genes involved in toxin detoxification are also reported in several carnivorous mammals, suggesting relaxed selection on those pathways in carnivores (Kim et al. 2016; Wagner et al. 2022).

Third, the amplification of specific gene copy numbers may be essential to enhance the absorption and synthesis of vital nutrients that are scarce in new diets (Závorka et al. 2023). For example, freshwater invertebrates generally lack docosahexaenoic acid (DHA), a polyunsaturated fatty acid essential for making cell membranes and lipid mediators (Twining et al. 2021). Therefore, freshwater fishes experience increased selective pressure to enhance their ability to synthesize DHA compared with marine fishes, which have access to DHA-rich prey (Ishikawa et al. 2019). An increase in the copy number and mRNA expression levels of the *fatty acid desaturase 2* (*Fads2*) gene, which is involved in the synthesis of DHA, has occurred repeatedly in freshwater fishes (Ishikawa et al. 2019). Thus, changes in gene copy number are often associated with diet shifts.

**Fig. 1.**
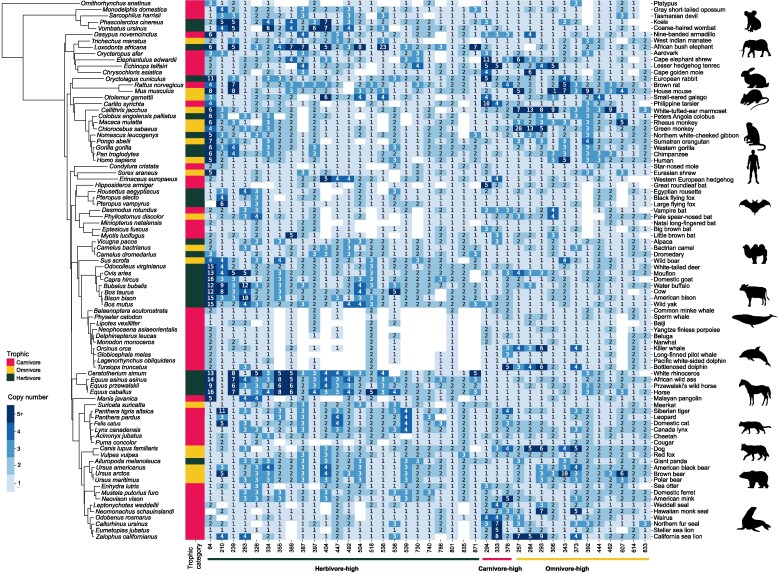
Gene families whose gene copy numbers are significantly associated with trophic categories (herbivore, carnivore, and omnivore) are mapped on a phylogenetic tree of mammals. The numbers at the bottom indicate the gene family IDs (refer to Table 1).

The present study aimed to identify candidate gene families whose copy numbers are associated with diet in mammals even after phylogenetic corrections and to test whether the copy numbers of specific gene families can be informative for inferring trophic ecology in 86 mammalian species analyzed here. Although previous studies have identified gene copy number changes associated with diet shifts in mammals, they have analyzed only specific genes (Zhang et al. 2002; Hughes et al. 2018), limited taxonomic groups (Walker and Roberts 2009; Kim et al. 2016; Rinker et al. 2019), or gene functional loss (Hecker et al. 2019; Wagner et al. 2022). Here, we conducted a genome-wide analysis of gene copy number variations using 86 mammalian species whose reference genome assemblies have gene annotations (Fig. 1 and supplementary table S1, Supplementary Material online). Additionally, to take phylogenetic signals into account, we employed a phylogenetic correction method (Hadfield and Nakagawa 2010). Given that closely related species often exhibit similar traits, e.g. gene copy number and trophic ecology in the present case, failure to incorporate phylogenetic corrections can lead to an overestimation of the significance of associations (Felsenstein 1985; Harvey and Pagel 1998). We also made a discriminant function (DF) that can distinguish between herbivores and carnivores and tested whether this function can predict the trophic ecology of mammals. We found 13 gene families whose copy numbers are useful for predicting trophic ecology in mammals.

## Results

### Gene Families Whose Gene Copy Numbers Are Positively Associated with Each Trophic Category

The median BUSCO scores of protein sequences annotated for 86 mammalian reference sequences analyzed were 97.9% (range = 84.4% to 99.4%) for the complete match and 98.6% (range = 93.9% to 99.6%) for the complete + partial matches (supplementary fig. S1, Supplementary Material online, and supplementary table S1, Supplementary Material online). Although carnivores tend to have relatively low BUSCO scores compared with herbivores and omnivores (supplementary fig. S1, Supplementary Material online), the differences among trophic categories were not statistically significant (Kruskal–Wallis test, *χ*^2^ = 2.343, *P* = 0.310 for the complete; *χ*^2^ = 3.484, *P* = 0.175 for the complete + partial).

Using the annotated protein sequences of these species, we first identified orthologs among protein sequences with SonicParanoid v1.3.8 (Cosentino and Iwasaki 2019). As the first screening for diet-associated gene copy numbers, we next selected genes that met all of the following criteria: (i) the ratio of the median copy number among species with a characteristic of interest (e.g. herbivory) divided by that among species without that characteristic (e.g. nonherbivory) is ≥2; (ii) 90% of species with the characteristic of interest have at least one copy; and (iii) 70% of species without the characteristic of interest have two or fewer copies. Although these criteria are arbitrary, the main aim of this step was not to identify all genes potentially important for diet shifts but to screen for genes that show strikingly convergent copy number changes. This screening identified 77, 19, and 61 gene families whose gene copy numbers are higher in herbivores, carnivores, and omnivores, respectively, than in other trophic categories (for the list of these genes, see doi:10.5061/dryad.q2bvq83r2).

**Fig. 2.**
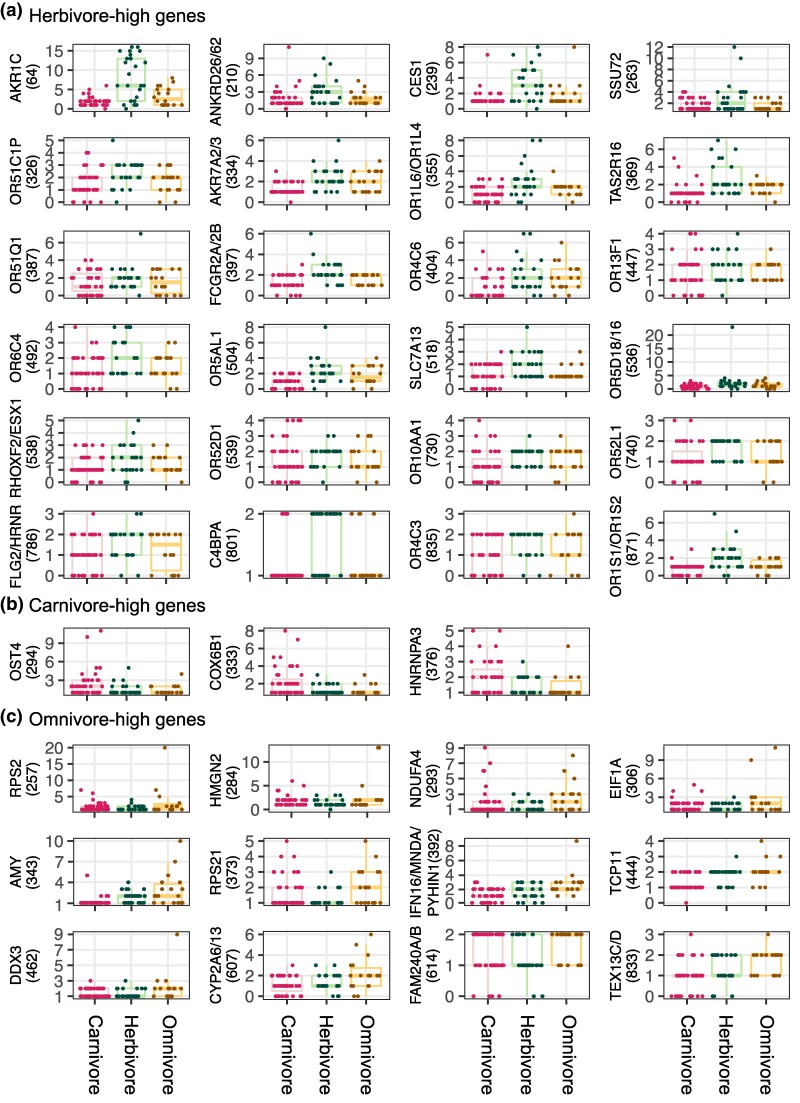
Copy number differences among trophic categories. Boxplots of copy numbers for genes whose copy numbers are higher in herbivores (a), carnivores (b), and omnivores (c) than in other trophic categories are shown. The numbers in parentheses are the gene family IDs (see Table 1). See supplementary fig. S3, Supplementary Material online, for a plot of gene family ID536 (OR5D16/18) after excluding one outlier herbivorous species (*Loxodonta africana*).

The gene copy numbers of these gene families were tested for the association with trophic category with phylogeny taken into account. We identified 24 gene families whose gene copy numbers are higher in herbivores than in nonherbivores at the level of *P* < 0.05 without Bonferroni corrections (Table 1, Figs. 1 and 2, and supplementary table S2, Supplementary Material online). After Bonferroni corrections, none were significant. Gene ontology (GO) analysis of 24 genes randomly selected per one gene family was repeated 10 times, and several GO terms appeared repeatedly as enriched GO terms (supplementary table S3, Supplementary Material online). GO terms related to aflatoxin B1 metabolism, olfaction, G protein-coupled receptor, and chemical perception appeared five or more times (supplementary table. S3, Supplementary Material online). GO term “aflatoxin B1 metabolism” is associated with gene families 64 (*AKR1C* genes), encoding aldo–keto reductase family enzymes, and 334 (*AKR7A2* and *AKR7A3*), encoding aflatoxin B1 aldehyde reductase. These are Phase II enzymes involved in detoxifying xenobiotic substances (Barski et al. 2008). Toxin detoxification generally starts with the oxidation of xenobiotic compounds (Phase I), which increases their water solubility and facilitates subsequent conjugation steps (Harborne 2014; Berg et al. 2015). In the next step (Phase II), xenobiotic compounds are conjugated with specific chemical groups, such as glutathione and glucuronides (Harborne 2014; Berg et al. 2015), which enhances the water solubility of xenobiotic compounds and allows them to be excreted through bile acids or urine. The copy numbers of genes encoding the enzymes catalyzing not only Phase II but also Phase I reactions, gene family 239 (*CES1*, encoding a liver carboxylesterase) (Ross and Crow 2007), were higher in herbivores (Table 1; Figs. 1 and 2). Among other herbivore-associated gene families, 13 gene families encode olfactory receptors, while one encodes a taste receptor (Table 1).

**Table 1:** Gene families whose gene copy numbers are positively associated with each trophic category

Trophic category	Gene family ID	Gene names in humans^$^	Possible functions of gene products	pMCMC	Pagel's *λ* (*P*-value)	Blomberg's *K* (*P*-value)
Herbivore	64^a^	*AKR1C4, AKR1C1, AKR1C2, AKR1C8, AKR1C3*	Aldo–keto reductase family 1 involved in Phase II detoxification (Barski et al. 2008)	0.0168	0.978 (<10^−24^)^c^	0.535 (0.001)^c^
Herbivore	210^a^	*ANKRD26, ANKRD62*	Ankyrin repeat domain-containing protein regulating centriole function (Evans et al. 2021); Causative gene for thrombocytopenia (Sullivan et al. 2022)	0.0204	0.336 (0.232)	0.049 (0.452)
Herbivore	239^a^	*CES1*	Liver carboxylesterase 1 involved in Phase I detoxification (Ross and Crow 2007)	0.03	0.961 (<10^−19^)^c^	0.317 (0.001)^c^
Herbivore	263	*SSU72*	Regulation of gene expression (Yeo et al. 2003)	0.0368	0.319 (0.028)	0.039 (0.664)
Herbivore	326	*OR51C1P*	Olfactory receptor	0.022	0.726 (0.339)	0.153 (0.001)^c^
Herbivore	334^a^	*AKR7A3, AKR7A2*	Aflatoxin B1 aldehyde reductase involved in Phase II detoxification (Barski et al. 2008)	0.01	0.802 (<10^−6^)^c^	0.094 (0.024)
Herbivore	355	*OR1L6, OR1L4*	Olfactory receptor	0.0252	0.893(<10^−10^)^c^	0.103 (0.027)
Herbivore	369	*TAS2R16*	Taste receptor	0.0372	0.984(<10^−13^)^c^	0.319 (0.001)^c^
Herbivore	387^a^	*OR51Q1*	Olfactory receptor	0.0248	0.928 (<10^−11^)^c^	0.185 (0.001)^c^
Herbivore	397^a^	*FCGR2A, FCGR2B*	Immune regulation (Smith and Clatworthy 2010)	0.002	0.730 (<10^−6^)^c^	0.128 (0.003)
Herbivore	404	*OR4C6*	Olfactory receptor	0.008	0.967 (<10^−12^)^c^	0.245 (0.001)^c^
Herbivore	447	*OR13F1*	Olfactory receptor	0.0476	0.662 (<10^−5^)^c^	0.071 (0.059)
Herbivore	492^a^	*OR6C4*	Olfactory receptor	0.0076	0.759 (<10^−8^)^c^	0.068 (0.057)
Herbivore	504^a^	*OR5AL1*	Olfactory receptor	0.0044	0.954 (<10^−7^)^c^	0.142 (0.003)
Herbivore	518	*SLC7A13*	Transport of aspartate, glutamate, and cysteine (Nagamori et al. 2016)	0.018	0.708 (<10^−7^)^c^	0.159 (0.001)^c^
Herbivore	536	*OR5D18, OR5D16*	Olfactory receptor	0.0464	0.996 (<10^−18^)^c^	0.489 (0.01)
Herbivore	538	*RHOXF2B, ESX1, RHOXF2*	Rhox homeobox family members involved in spermatogenesis (Niu et al. 2011)	0.0172	0.306 (0.114)	0.043 (0.464)
Herbivore	539	*OR52D1*	Olfactory receptor	0.026	0.521(<10^−4^)^c^	0.091 (0.006)
Herbivore	730^a^	*OR10AA1, OR10AA1C* ^b^	Olfactory receptor	0.004	1.002 (<10^−16^)^c^	0.385 (0.001)^c^
Herbivore	740	*OR52L1*	Olfactory receptor	0.0044	0.627 (<10^−3^)^c^	0.081 (0.009)
Herbivore	786^a^	*FLG2, HRNR*	Skin barrier function (Brown et al. 2012)	0.0264	7.35 × 10^−5^ (1)	0.045 (0.237)
Herbivore	801	*C4BPA*	Complement inhibitor (Ermert and Blom 2016)	0.0248	1.002 (<10^−12^)^c^	0.323 (0.001)^c^
Herbivore	835	*OR4C3*	Olfactory receptor	0.008	1.002 (<10^−21^)^c^	0.503 (0.001)^c^
Herbivore	871^a^	*OR1S1, OR1S2*	Olfactory receptor	0.002	0.991 (<10^−10^)^c^	0.300 (0.001)^c^
Carnivore	294	*OST4*	Dolichyl-diphosphooligosaccharide–protein glycosyltransferase involved in the clearance of advanced glycation end products (Zhuang et al. 2017)	0.028	0.965 (<10^−8^)^c^	0.195 (0.014)
Carnivore	333^a^	*COX6B1*	Stabilization of cytochrome c oxidase dimers (Yoshikawa et al. 1998)	0.008	0.142 (0.274)	0.101 (0.054)
Carnivore	376^a^	*HNRNPA3*	Component of 40S ribosomal subunits (Plomaritoglou et al. 2000)	0.024	0.087 (0.465)	0.024 (0.933)
Omnivore	257	*RPS2*	Component of ribosome	0.025	0.068 (0.576)	0.095 (0.349)
Omnivore	284	*HMGN2*	Regulation of gene expression (He et al. 2018)	0.018	0.125 (0.250)	0.111 (0.18)
Omnivore	293	*NDUFA4*	Component of the cytochrome c oxidase	0.0464	7.35 × 10^−5^ (1)	0.035 (0.661)
Omnivore	306	*EIF1AX, EIF1AY*	Regulation of translation (Hinnebusch and Lorsch 2012)	0.007	0.165 (0.527)	0.1052 (0.179)
Omnivore	343	*AMY1A, AMY1B, AMY1C, AMY2A, AMY2B*	Alpha-amylase involved in starch digestion (Pajic et al. 2019)	<0.0001	0.489 (0.019)	0.057 (0.417)
Omnivore	373	*RPS21*	Component of the 40S subunit	0.031	0.095 (0.416)	0.034 (0.684)
Omnivore	392	*IFN16, MNDA, PYHIN1*	Transcriptional regulation in myeloid cell (Gu et al. 2022)	0.023	0.678 (<10^−9^)^c^	0.206 (0.001)^c^
Omnivore	444	*TCP11, TCP11X1, TCP11X2*	Regulation of sperm motility (Castaneda et al. 2020)	0.001	0.775 (<10^−6^)^c^	0.096 (0.009)
Omnivore	462	*DDX3X, DDX3Y*	Antiviral function (Khadivjam et al. 2017)	0.011	0.115 (0.720)	0.088 (0.313)
Omnivore	607	*CYP2A6, CYP2A13*	Cytochrome P450 involved in Phase I detoxification (Murayama and Yamazaki 2021)	0.030	0.451 (0.001)^c^	0.096 (0.02)
Omnivore	614	*FAM240A, FAM240B*	unknown	0.016	0.253 (0.028)	0.066 (0.052)
Omnivore	833	*TEX13C, TEX13D*	unknown	0.028	0.423 (0.001)^c^	0.045 (0.392)

^a^Thirteen gene families used for the final model of DF after a stepwise variable selection are marked. Gene family ID corresponds to the number at the bottom of Fig. 1.

^b^Gene names are based on *Homo sapiens* except gene family 730 (*OR10AA1* and *OR10AA1C*), whose names are based on *B. taurus*, because humans lack that gene family.

^c^Significant after the Bonferroni corrections: we conducted corrections because we conducted 39 statistical tests with 39 gene families.

Land plants generally lack DHA, while prey animals contain DHA (Twining et al. 2021). Therefore, we hypothesized that herbivores might have higher copy numbers of *FADS* gene families than carnivores to enhance DHA synthetic abilities. However, copy numbers of *FADS* were not significantly different among trophic categories (supplementary fig. S2, Supplementary Material online, and supplementary table S4, Supplementary Material online), suggesting that DHA deficiency in land plants is not a major constraint of a shift to herbivory.

In contrast to herbivore-high genes, we found only three carnivore-high gene families (*OST4*, *COX6B1*, and *HNRNPA3*) at the level of *P* < 0.05 with no significant genes after Bonferroni corrections (Table 1; Fig. 2b). None of these genes have been previously reported to be associated with diets.

Twelve gene families showed higher copy numbers in omnivores than in herbivores and carnivores at *P* < 0.05 (Table 1), although none were significant after Bonferroni corrections. A previous study showed that mammals consuming a broad range of diets have a higher copy number of *AMY* genes than herbivores and carnivores (Pajic et al. 2019). As we found gene family 343, containing genes encoding amylases, in the candidate omnivore-high gene families, we could confirm this trend in a much larger dataset, although the association was not significant after Bonferroni corrections. GO analysis of 12 omnivore-associated genes randomly selected per one gene family was repeated 10 times, and GO terms related to ribosome and translation appeared five or more times as enriched terms (supplementary table. S5, Supplementary Material online).

Copy number increases can occur by either tandem or interchromosomal duplication (Groot et al. 1990). We analyzed the location of duplicated genes. Many herbivore-high genes showed tandem duplications, whereas no carnivore-high genes showed tandem duplications (Table 2 and supplementary tables S6 and S7, Supplementary Material online). Omnivore-high genes showed intermediate patterns with both tandem and interchromosomal duplications (Table 2 and supplementary table S8, Supplementary Material online).

**Table 2:** Locations of duplicated genes

Types of genes	Species	Tandem	Interchromosomal	Both	ND
Herbivore-high	*Ailuropoda melanoleuca*	5	0	1	18
Herbivore-high	*Bos taurus*	15	3	3	3
Herbivore-high	*Equus caballus*	13	1	1	9
Herbivore-high	*Gorilla gorilla*	6	3	1	14
Herbivore-high	*Loxodonta africana*	9	2	5	8
Herbivore-high	*Nomascus leucogenys*	9	2	1	12
Herbivore-high	*Oryctolagus cuniculus*	10	0	2	12
Carnivore-high	*Desmodus rotundus*	0	1	0	2
Carnivore-high	*Eptesicus fuscus*	0	1	0	2
Carnivore-high	*Felis catus*	0	2	0	1
Carnivore-high	*Rattus norvegicus*	0	2	0	1
Carnivore-high	*Zalophus californianus*	0	3	0	0
Omnivore-high	*Callithrix jacchus*	1	5	2	4
Omnivore-high	*Canis lupus familiaris*	2	6	0	4
Omnivore-high	*Homo sapiens*	4	3	1	4
Omnivore-high	*Macaca mulatta*	1	7	1	3
Omnivore-high	*Mus musculus*	4	4	2	2
Omnivore-high	*Phyllostomus discolor*	2	5	0	5
Omnivore-high	*Sorex araneus*	2	3	1	6
Omnivore-high	*Suricata suricatta*	1	3	0	8

ND, not determined because any contig with a gene is not anchored to a specific chromosome or the gene is absent.

### Inference of Trophic Ecology From Gene Copy Numbers

**Fig. 3.**
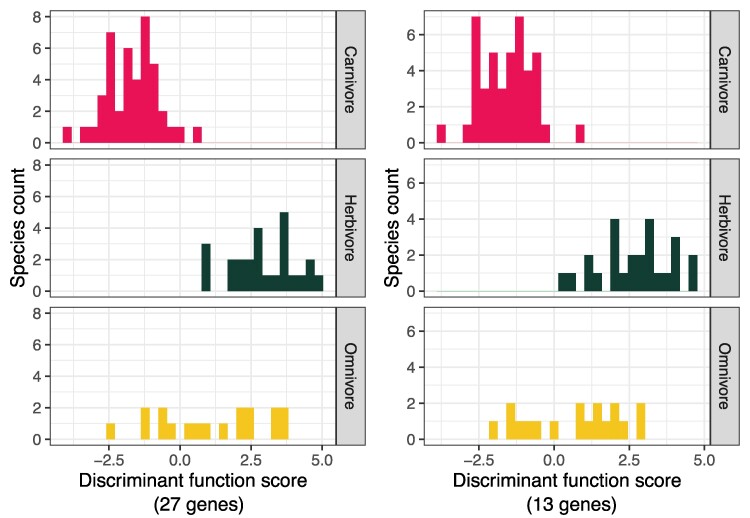
Histogram of DF scores calculated from a linear combination of the weighted values of gene copy numbers (supplementary tables S7 and S9, Supplementary Material online). (a) Twenty-seven gene families identified in this study were used for making the function. (b) Thirteen gene families selected by a stepwise variable selection were used. For DF scores of each species, see supplementary tables S8 and S10, Supplementary Material online.

To investigate whether we can distinguish between herbivores and carnivores using gene copy numbers, we first conducted a linear DF analysis using the gene copy numbers of 27 gene families that are higher in either herbivores or carnivores at *P* < 0.05 without Bonferroni corrections (Table 1). The DF distinguished between herbivores and carnivores (Fig. 3a; supplementary tables S7 and S8, Supplementary Material online). Leave-one-out cross-validation showed that the predictive accuracy of the model with 27 gene families was 88.2%. Four carnivorous species, *Condylura cristata*, *Manis javanica*, *Physeter catodon*, and *Rattus norvegicus*, were classified as herbivores. Although *R. norvegicus* was classified as a carnivore in our initial dataset, which we call Dataset 1, it can also feed on plants (Guiry and Buckley 2018). Four herbivorous species, *Loxodonta africana*, *Ovis aries*, *Pteropus alecto*, and *Vicugna pacos*, were classified as carnivores. Calculation of scores with this DF in omnivores showed that omnivores showed a broad range of DF scores (Fig. 3a and supplementary table S10, Supplementary Material online).

Using a stepwise variable selection, we reduced the number of gene families for making a DF to 13 (Fig. 3b; supplementary tables S11 and S12, Supplementary Material online): Wilks’ *λ* of this model was 0.186, which was slightly higher than that with all 27 genes (Wilks’ *λ* = 0.161). These selected 13 gene families are marked with asterisks in Table 1. Leave-one-out cross-validation showed that the predictive accuracy of the model with 13 gene families was 91.1%. Among 43 carnivores, 41 species were successfully classified as carnivores with *C. cristata* and *R. norvegicus* misclassified. Among 25 herbivores, 21 species were correctly classified as herbivores with *O. aries*, *P. alecto*, *P. vampyrus*, and *V. pacos* misclassified.

To further investigate how uneven taxon sampling can influence the predictive ability of DF with the gene copy numbers of the 13 gene families, we left out one clade for making a DF and tested whether the trophic ecology of the species belonging to the removed clade could be predicted by that DF. When we made a DF without *Chiroptera*, which included five carnivores, one omnivore, and three herbivores, we could successfully predict the trophic ecology of five carnivores and three herbivores with one omnivore (*Phyllostomus discolor*) being predicted as a carnivore. When we left out Cetacea, which included 10 carnivores, and made a DF, all 10 cetacean species were predicted as carnivores. These results suggest that the gene copy numbers of these 13 gene families are useful for predicting the trophic ecology to some extent.

### Analysis of Phylogenetic Signals

Although we screened for gene families whose gene copy numbers are associated with the trophic category after phylogenetic corrections at the level of *P* < 0.05 without Bonferroni corrections (Table 1), it does not mean that the gene copy numbers evolve independently of the phylogeny. To investigate how the phylogeny influences the gene copy numbers, we calculated Pagel's *λ* and Blomberg's *K* (Pagel 1999; Blomberg et al. 2003) (Table 1). In both indexes, 0 indicates the absence of phylogenetic bias, while 1 indicates a strong bias in accordance with the model of Brownian motion along the phylogeny. We found Pagel's *λ* of 20 herbivore-high, 1 carnivore-high, and 6 omnivore-high gene families was larger than 0 at *P* < 0.05, while 19, 1, and 4 gene families remained significant after Bonferroni corrections. Blomberg's *K* of 18 herbivore-high, 1 carnivore-high, and 3 omnivore-high gene families was significantly larger than 0 at *P* < 0.05, while only 11, 0, and 1 gene families remained significant after Bonferroni corrections.

Because the evolution of the gene copy numbers is substantially influenced by the phylogeny and DF analysis in the previous section did not consider phylogeny, we next employed phylogenetic discriminant analysis. The optimal *λ* value was 0, indicating that the effects of phylogeny on the relationships between the trophic category and the gene copy numbers are small. When we used different values of *λ* ranging from 0 to 0.1, the misclassification rates ranged from 0.029 to 0.044, with *V. pacos* and *C. cristata* being always misclassified (supplementary tables S13, Supplementary Material online), *R. norvegicus* misclassified at *λ* = 0, and *P. vampyrus* misclassified at *λ* = 0.1. These results suggest that the gene copy numbers of 13 gene families are useful for predicting the trophic category to some extent regardless of whether the phylogeny is taken into account.

Overall, these results indicate that the copy number of each gene family evolves under the influence of phylogeny. However, combining the information from 13 gene families, we can predict the trophic ecology to some extent.

### Caveats of Categorical Classification of Trophic Ecology

The categorical classification of herbivores, carnivores, and omnivores is very simplistic, as several species do not fit perfectly into one of them. For example, *Ursus maritimus* can be classified as a carnivore, *Trichechus manatus* and *Camelus bactrianus* as herbivores, and *Monodelphis domestica*, *Pan troglodytes*, *Ailuropoda melanoleuca*, and *R. norvegicus* as omnivores (Kissling et al. 2014; Samuels 2009). We changed the trophic categories of these seven species, which we call Dataset 2 (supplementary table S1, Supplementary Material online), and conducted DF analysis with the 13 genes (supplementary tables S14, Supplementary Material online). We made a new DF using Dataset 2, and the new DF classified 41 carnivores as carnivores (97.6%) with only one carnivore, *C. cristata*, being misclassified as a herbivore. Among 25 herbivores, 22 species (88%) were classified as herbivores with three species, *P. alecto*, *T. manatus*, and *V. pacos*, classified as carnivores. Phylogenetic DF analysis using this Dataset 2 also showed that the optimal *λ* = 0. Varying *λ* from 0 to 0.1, the misclassification rates were always 0.045 with *V. pacos* predicted as an herbivore and *C. cristata* and *T. manatus* as carnivores. Thus, these analyses overall showed that the copy numbers of these 13 genes are informative for predicting the trophic ecology, although we cannot exclude the possibility that the prior simple categorization of trophic ecology can influence the predictability to some extent.

**Fig. 4.**
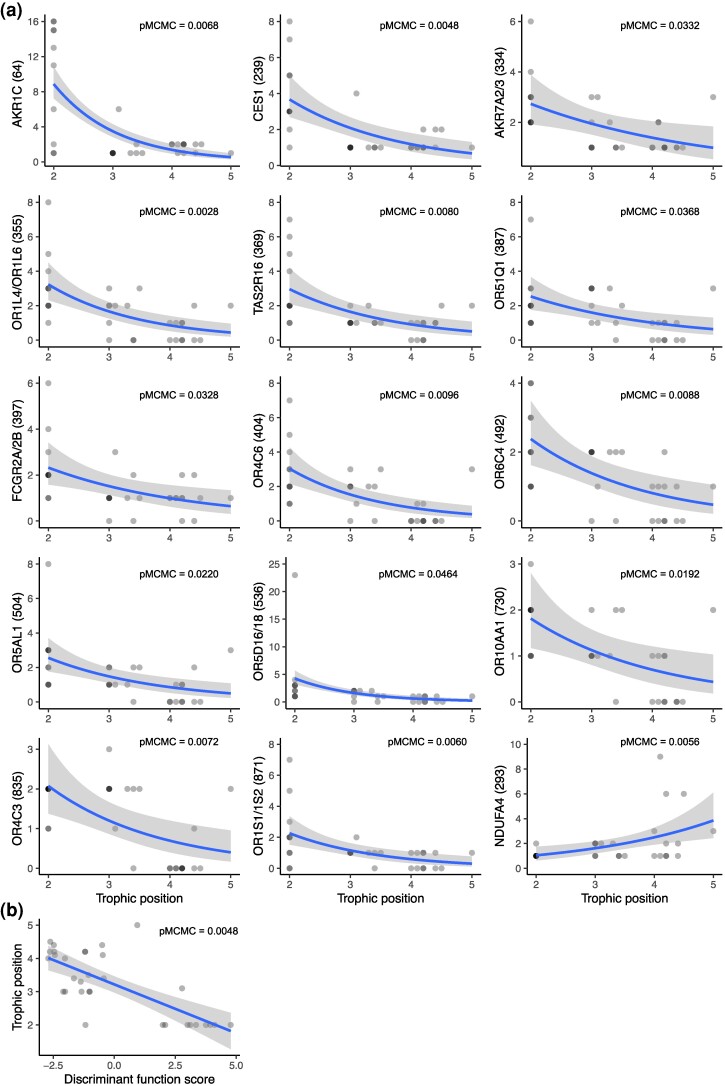
The association between gene copy number and trophic level. a) Genes whose copy numbers are associated with the quantitative measurements of trophic levels at the level of *P* < 0.05 without Bonferroni corrections are shown. Poisson regression lines (blue lines) and standard errors (gray areas) are also shown. The numbers in parentheses are the gene family IDs (see Table 1). b) DF scores calculated with 13 genes were significantly associated with the quantitative measurements of trophic levels. Linear regression lines and 95% confidence bands are also shown.

Finally, to avoid the issue of simple categorical classification, we tested whether the copy numbers of any of these gene families are significantly associated with quantitative measurements of trophic levels. The quantitative measurements of trophic levels were available for 32 species (supplementary tables S15, Supplementary Material online) (Tucker and Rogers 2014). After taking phylogeny into consideration, copy numbers of 14 herbivore-high gene families and one omnivore-high gene family in Table 1 turned out to be associated with the trophic levels at *P* = 0.05 (Fig. 4a), although none were significant after Bonferroni corrections. The DF score calculated with 13 genes (see the previous section) was also significantly associated with the trophic level (pMCMC = 0.0048), consistent with the idea that this DF score can be a predictor of trophic ecology (Fig. 4b).

## Discussion

### Gene Copy Number Changes Associated with Diets in Mammals

We identified several gene families whose gene copy numbers are associated with diets in mammals. Our screening successfully identified previously reported genes associated with diets. First, we confirmed that omnivores have a higher copy number of amylase genes than herbivores and carnivores using a larger dataset than those of previous studies (Pajic et al. 2019). Second, higher copy numbers of several olfactory receptors in herbivores, previously reported in Hughes et al. (2018) and Policarpo et al. (2024), have also been confirmed in our study. Previous studies reported the expansion of olfactory receptors in herbivores and suggested that increases in these genes may be related to increased efficiency in detecting digestible plants (Hughes et al. 2018; Policarpo et al. 2024).

We also demonstrated higher copy numbers of several genes encoding enzymes involved in the detoxification of xenobiotic substances (Table 1) in herbivores. Previous analysis of 18 mammalian species, all of which except the lion were included in our analysis, showed that genes encoding the Uridine diphosphate-glucuronosyltransferase, a Phase II enzyme, had lower copy number in carnivores (Kim et al. 2016). A previous analysis of gene functional loss using 31 mammals showed the loss of genes encoding xenobiotic receptors (*NR1I3* and *NR1I2*) in carnivores (Hecker et al. 2019). These findings suggest that the detoxification of xenobiotic substances plays a crucial role in the consumption of plants. As plants produce secondary metabolites as a defense mechanism, these copy number variations likely reflect signatures of an arms race between plants and plant-eating animals (Freeland and Janzen 1974).

We also found several new candidate genes whose copy numbers are associated with diets. We found that omnivores have higher copy numbers of several genes encoding transcriptional or translational regulators, such as *RPS2*, *RPS21*, *HMGN2*, *EIF1AX*, *EIF1AY*, *IFN16*, *MNDA*, and *PYHIN1* genes. Diets generally change the expression of genes involved in metabolism as a plastic response (Clarke and Abraham 1992; Jousse et al. 2004; Salati et al. 2004). Protein translation is also regulated by diet and nutritional status (Proud 2002; Shu et al. 2020). Because omnivores eat a wide range of diets, higher copy numbers of these genes may be adaptive for changing gene expression and mRNA translation depending on their dietary intake.

We found carnivores to have higher copy numbers of the *COX6B1* gene. Cytochrome c oxidase 6B1 stabilizes the dimerization of cytochrome c oxidase (Yoshikawa et al. 1998), and up-regulation of Cytochrome c oxidase 6B1 is protective against oxidative stress in neurons (Yang et al. 2019). Therefore, increased copy numbers of the *COX6B1* may up-regulate the expression levels of Cytochrome c oxidase 6B1 and contribute to tolerance against mitochondrial oxidative stress induced by meat-derived iron. Carnivores also had a higher copy number of the *OST4* gene encoding dolichyl-diphosphooligosaccharide–protein glycosyltransferase, which is involved in the clearance of advanced glycation end products (AGEs) (Zhuang et al. 2017). Because meats generally contain higher amounts of AGEs than vegetables (Uribarri et al. 2010) and AGEs can induce inflammation (Yan et al. 2008), a higher copy number of *OST4* may be adaptive for clearance of AGEs. However, this is only speculation, and further experiments on the roles of these genes in diet adaptation are necessary.

### Modes of Gene Duplication

Carnivore-high genes tended to be located on different chromosomes, indicating that these genes are duplicated by interchromosomal duplication. In contrast, many herbivore-high genes are located close to each other on the same chromosome, indicating that tandem duplication occurred in these genes. A recent study demonstrated an accumulation of DNA transposons among carnivorous lineages compared to herbivorous counterparts (Osmanski et al. 2023). The increased activities of transposons might promote interchromosomal duplication in carnivores. Although we do not know why genes whose copy numbers are positively associated with different trophic categories are duplicated in different modes, a previous genetic manipulation study on an alcohol dehydrogenase gene in *Drosophila* showed that a tandem duplication has a larger effect on gene expression than an interchromosomal duplication (Loehlin and Carroll 2016). Furthermore, tandemly duplicated genes tend to share the same *cis*-regulatory elements, while interchromosomal transposition can put the duplicated copies under the control of different *cis*-regulatory elements (Arthur et al. 2014). Therefore, tandem duplication may be more efficient for increasing the overall gene expression levels, whereas interchromosomal duplication may be more efficient for causing subfunctionalization. Further studies on the gene expression patterns of duplicated genes identified in the present study will contribute to a better understanding of the functions of different modes of gene duplications in diet adaptation.

Mutation rates vary across the genome (Hodgkinson and Eyre-Walker 2011; Makova and Hardison 2015). If certain genes have higher mutation rates than others, mutations at these genes are more likely to be captured by natural selection. Genes that are repeatedly used for convergent evolution sometimes have higher mutation rates (Storz et al. 2019; Xie et al. 2019). Currently, we do not know whether the identified 13 gene families have higher background copy number mutation rates compared to other genes. It is important to investigate whether the genomic loci of these 13 gene families have any characteristics of mutation hotspots (Hodgkinson and Eyre-Walker 2011; Makova and Hardison 2015).

### Prediction of Trophic Ecology, Caveats, and Future Directions

We have shown that copy numbers of a specific set of genes may be useful for predicting the trophic ecology of a mammal. Such information could be useful for predicting whether a particular animal can be raised in captivity with plant-derived diets. If carnivores and omnivores exhibit DF scores close to those of herbivores, they may be able to thrive on plant-derived diets in a livestock setting. Currently, an increasing number of mammalian genome sequences are being determined (Zoonomia Consortium 2020; Christmas et al. 2023), which will reduce the caveat of uneven sampling and further improve the predictive power to infer animal ecology from genome sequence data.

There are several important caveats in the present study and a lot of room for further improvement. First, classification of species into three trophic categories is oversimplification. For example, carnivores can be further classified into subcategories based on their diets, for example, sanguivores, insectivores, and piscivores. We may overlook genes whose copy numbers are specifically associated with a particular subcategory of diet. As whole-genome sequences of more species are available (Zoonomia Consortium 2020; Christmas et al. 2023), it will become possible to analyze gene copy numbers associated with the subcategory of trophic ecology. Additionally, quantitative measurements of trophic levels rather than categorical classification may be useful for finding genes or gene families whose copy numbers are associated with diets. However, because observations of natural diets do not necessarily provide information on the obligate or opportunistic characteristics, investigation of diets in nature and captivity will improve the accuracy of a predictive model of diets. The present study also overlooked interindividual and interpopulation variations in diets within species. Therefore, analysis of potential associations between diet compositions and copy number variation within species is also necessary in the future.

Second, because we took a macroevolutionary approach, we are likely to overlook clade-specific mechanisms for dietary adaptation. In the family Ursidae, for example, the DF score with 13 genes of herbivorous giant panda (*A. melanoleuca*; 1.964) was close to the omnivorous American black bear (*Ursus americanus*; 1.977), although the brown bear (*Ursus arctos*; 1.300) and the polar bear (*U. maritimus*; 0.925) had relatively low scores. Detailed comparative analysis of genomes within specific clades is necessary for identifying clade-specific mechanisms. Furthermore, comparison of closely related species that differ in the diets will enable to tell whether and when a gene copy number increased or decreased.

Third, incomplete protein annotation may bias our results. Although we found that the BUSCO scores were high for the species analyzed, there was a trend of carnivores having lower scores compared to other categories. Because annotation heterogeneity can bias the result of lineage-specific genes (Weisman et al. 2022), further improvement of genome sequences and annotations will increase the accuracy of a predictive model.

Fourth, our analysis is based on a single reference species tree. However, different parts of the genome can differ in their evolutionary histories due to incomplete lineage sorting, introgression, and gene conversion between paralogs (Hahn 2019). If some traits, such as trophic ecology or gene copy numbers, evolve along a gene tree discordant with the species tree, our phylogenetic comparative methods can be erroneous (Hibbins et al. 2023). As the method incorporating gene tree histories into comparative methods is being developed (Hibbins et al. 2023), it will be necessary to test how gene tree discordances influence our results in the future.

Fifth, we did not compare amino acid sequences or gene expression patterns among duplicated copies. Although an increase in gene copy number can increase the expression level of a gene and its paralogs overall (Henrichsen et al. 2009; Orozco et al. 2009), a duplicated gene sometimes acquires a new function in both gene expression patterns and protein functions (Ohno 1970). Further analysis of functional divergence among duplicated copies is necessary for a better understanding of the roles of these gene duplications in diet shifts. Additionally, it is possible that genes acquire new functions solely by protein sequence changes without any gene duplication (Matsushita et al. 2020).

Finally, our present study is based on bioinformatic analysis. Experimental validation of the adaptive significance of gene copy number changes in the utilization of different diets is necessary using any genetically tractable systems, such as mice.

## Conclusion

In summary, we found 13 gene families whose copy numbers repeatedly change in association with diet shifts and are useful for predicting trophic ecology in our datasets of 86 mammalian species. Further studies of the functions and mutation rates of these genes will provide great insight into the ecological and genetic mechanisms underlying convergent evolution. Our results also indicate the possibility that genome sequence data can be used to develop methods for inferring an animal's trophic ecology and ability to adapt to a new diet. Currently, whole-genome sequences are being determined for an increasing number of organisms. With such increasing genome sequence information, it will be possible to test the robustness of the predictive model and further improve it in the near future.

## Materials and Methods

### Screening for Candidate Genes Whose Copy Numbers Are Associated with Diets

For searching for candidate genes, we used 86 mammalian species (Fig. 1 and supplementary table S1, Supplementary Material online) that are highly studied representatives of mammalian diversity and also had reference genome assemblies with gene annotations (for accession numbers, see supplementary table S1, Supplementary Material online). After downloading the annotation file in GenBank format, we first selected only coding sequences (CDSs) that have “protein_id” and “translation” qualifiers. When multiple CDSs existed for the same Entrez Gene ID, we selected the longest protein. We next identified orthologs among these protein sequences using SonicParanoid v1.3.8 with default parameters in the default mode (Cosentino and Iwasaki 2019). SonicParanoid is a software program for identifying orthologous relationships among multiple species (Cosentino and Iwasaki 2019). For assessing the completeness of the annotated proteins, we calculated the BUSCO scores using the protein sequences analyzed (supplementary table S1, Supplementary Material online) with the “eukaryota” subset of BUSCO v5 (Manni et al. 2021). For each gene family in Table 1, the *Homo sapiens* gene names included in each family were extracted from the GenBank annotation file, with the exception of gene family 730, for which no *H. sapiens* genes are present. In the case of gene family 730, the gene names included in this family were derived from *Bos taurus*.

We next collected data on the categorical classification (herbivores, carnivores, and omnivores) of 86 species from the Dryad database (doi:10.5061/dryad.qd450) (Gainsbury et al. 2018). For the species whose trophic categories are not listed in the database, we first searched the Animal Diversity Web (https://animaldiversity.org/), constructed by the University of Michigan. For three species not included in the second database, we looked into published papers: *Neophocaena asiaeorientalis* (Lu et al. 2016) and *T. manatus* (Courbis and Worthy 2003). This dataset (Dataset 1) used in this study is available in supplementary table S1, Supplementary Material online.

As the first screening for diet-associated gene copy numbers, we selected genes that met all of the following criteria: (i) the ratio of the median copy number of species with a characteristic of interest (e.g. herbivory) divided by that of species without that characteristic (e.g. nonherbivory) is ≥2; (ii) 90% of species with the characteristic of interest have at least one copy; and (iii) 70% of species without the characteristic of interest have two or fewer copies. To obtain *FADS* 1 to 4 copy number data from multiple mammalian species, we used a web tool ORTHOSCOPE v.1.0.2 (Inoue and Satoh 2019) as described previously (Ishikawa et al. 2019).

Because the inclusion of pseudogenes increases phylogenetic signals and may reduce the association between gene copy number and trophic ecology, we examined whether the orthologous genes in Table 1 contained pseudogenes. Only human *OR51C1P* (*Olfactory Receptor Family 51 Subfamily C Member 1 Pseudogene*: NP_001382980.1) had the annotation of “pseudogene” in its gene name. However, *OR51C1P* has an open reading frame encoding 312 amino acids, and the RefSeq status was “Validated” as a protein-coding gene (https://www.ncbi.nlm.nih.gov/gene/401661).

### Phylogenetic Corrections and Phylogenetic Signals

To ensure that the observed correlations between gene copy number and diet were not solely a product of phylogenetic relatedness, we employed a Bayesian inference approach using a generalized linear mixed model (GLMM), as described previously (Ishikawa et al. 2022). This analysis accounted for phylogeny as a covariate, utilizing the MCMCglmm R package (Hadfield and Nakagawa 2010) in conjunction with a mammalian phylogenetic tree downloaded from the Dryad database (global RAxML tree: doi.org/10.5061/dryad.tb03d03) (Upham et al. 2019). The estimated copy number of each gene was treated as the response variable, while the diet type (herbivory, carnivory, or omnivory) or the trophic level was used as the predictor variable. We used an inverse Wishart prior (parameters *V* = 1 and *ν* = 0.002), and all models were run for 1,050,000 iterations with a burn-in of 50,000 iterations and a thinning interval of 200. We also calculated the phylogenetic signals, the Pagel's *λ* and the Blomberg's *K* (Pagel 1999; Blomberg et al. 2003), of gene copy numbers using phylosig implemented in phytools (Revell 2012).

### Analysis of GO and the Locations of Duplicated Genes

To characterize the functions of genes whose copy numbers are associated with diets, we conducted GO analysis. The genes and associated GO terms of herbivorous *B. taurus* (cow) and omnivorous *H. sapiens* (humans) were used for the analysis. One gene was randomly selected per one gene family and used for the analysis. Random selection was repeated 10 times, and GO analysis was performed with 10 different sets of randomly selected genes using an R statistical package “gprofiler2” with default settings (Kolberg et al. 2020). As we found only three carnivore-associated gene families (see Results), we did not conduct GO analysis of these carnivore-high genes.

To investigate whether gene duplication occurred by intrachromosomal tandem duplication or interchromosomal gene duplication, we downloaded the chromosomal position data from the NCBI genome database (https://www.ncbi.nlm.nih.gov/genome/). For each trophic category, we selected species whose genomes are assembled into chromosomes (Table 2). When the duplicated genes are located within 5 Mb on the same chromosomes, we classified them into intrachromosomal tandem duplications. When located on different chromosomes or >5 Mb away from another copy, they were classified into interchromosomal gene duplications. When a species had no copy or just one copy, that gene was classified as “not-determined” in that species. When a gene is located on a contig that cannot be anchored to a specific chromosome (ChrUn in the NCBI database), we classified them into “not-determined” as well.

### DF Analysis

To investigate whether we can distinguish between herbivores and carnivores using gene copy numbers of particular gene families, we first conducted a linear DF analysis using the copy numbers of 24 herbivore-high gene families and 3 carnivore-high gene families (Table 1). We used the lda function in the R package MASS (Venables and Ripley 2002) to find a function distinguishing carnivores and herbivores best. Using a function that maximizes the ratio of between-group variation to within-group variation, we calculated DF scores for all 86 mammals analyzed in this study. To reduce the number of gene families for the DF, we next performed a stepwise forward variable selection using Wilks’ *λ* criterion with the R package klaR with default settings (Weihs et al. 2005). To validate the DF with the initial 27 gene families and the selected 13 gene families, we conducted leave-one-out cross-validation implemented in MASS. To test whether the removal of a particular clade can influence the predictive accuracy, we made a DF without a particular clade and tested whether the trophic categories of the removed species can be predicted. We removed nine species of Chiroptera (five carnivores, one omnivore, and three herbivores) in one test and 10 species of Cetacea (10 carnivores) in another test. R scripts used for the DF analysis are available from Dryad (doi:10.5061/dryad.q2bvq83r2).

Discriminant analysis with phylogeny taken into account was conducted using phylo.fda.v0.2.R (https://github.com/lschmitz/phylo.fda/blob/master/phylo.fda.v0.2.R) (Motani and Schmitz 2011; Schmitz and Motani 2011). Before analysis, the global RAxML tree used for the phylogenetic corrections was transformed into an ultrametric tree using chronos (Sanderson 2002; Kim and Sanderson 2008; Paradis 2013) in ape v5.7.1 (Paradis and Schliep 2019). We first calculated the optimal *λ* value using copy numbers of 13 gene families of 43 carnivores and 25 herbivores. Although we found that optimal *λ* value = 0, we conducted discriminant analysis with *λ* values ranging from 0 to 0.1 with a step size of 0.01. The proportions of the training data were used as the prior probabilities: carnivore/herbivore = 0.632:0.368.

### Analysis with Different Trophic Categories and Quantitative Trophic Levels

Because different literatures classify several species into different trophic categories, we tested whether the copy numbers of the selected 13 gene families are informative for predicting the diets even after changing the trophic categories of several species. To this end, we conducted new DF analyses after changing the trophic categories of Dataset 1 into the following categories: *U. maritimus* as a carnivore; *T. manatus* and *C. bactrianus* as herbivores; *M. domestica*, *P. troglodytes*, *A. melanoleuca*, and *R. norvegicus* as omnivores, following (Samuels 2009; Price et al. 2012) and the Animal Diversity Web. Using this new dataset (Dataset 2), we made new discriminant functions with 13 gene families. This dataset (Dataset 2) is available in supplementary table S1, Supplementary Material online.

We also tested whether the copy numbers of the selected 13 gene families are associated with the quantitative measurement data of trophic level. Quantitative measurement data of trophic level were obtained from a previous paper (Tucker and Rogers 2014). Primary producers, such as plants, are located at the trophic level 1, and herbivores are assigned to the trophic level 2. In Tucker and Rogers (2014), the trophic level of carnivore *i* (TL*_i_*) was determined using the following equation:


$$TL_{i}=1+\;\left(\frac{\sum_{\;j=1}^{n}\;DC_{ij}TL_{j}}{\sum_{\;j=1}^{n}\;DC_{ij}}\right),$$

where DC*_ij_* is the diet composition with the proportion of prey *j* in the diet of species *i*, *n* is the number of prey groups, and TL*_j_* is the trophic level of prey *j* with TL*_j_* = 2 for herbivorous prey species, TL*_j_* = 2.5 for omnivorous prey species, and TL*_j_* = 3 for carnivorous prey species. As described above, we tested whether the trophic level can be a predictor of the copy number of each gene family using a phylogeny-corrected GLMM with a Poisson model, utilizing the MCMCglmm R package.

## Supplementary Material

evaf008_Supplementary_Data

## Data Availability

Codes used in this study are available from Dryad doi: doi:10.5061/dryad.q2bvq83r2. Other raw data are available as supplementary tables.
