# Supplementary material for: Convergent Evolution and Predictability of Gene Copy Numbers Associated with Diets in Mammals
Source: Genome Biol Evol. 2025 Jan 24;17(2):evaf008. doi: 10.1093/gbe/evaf008 (PMC11797053; doi:10.1093/gbe/evaf008)
Supplement: evaf008_Supplementary_Data [file evaf008_supplementary_data.zip › Supplementary_figures.pdf]

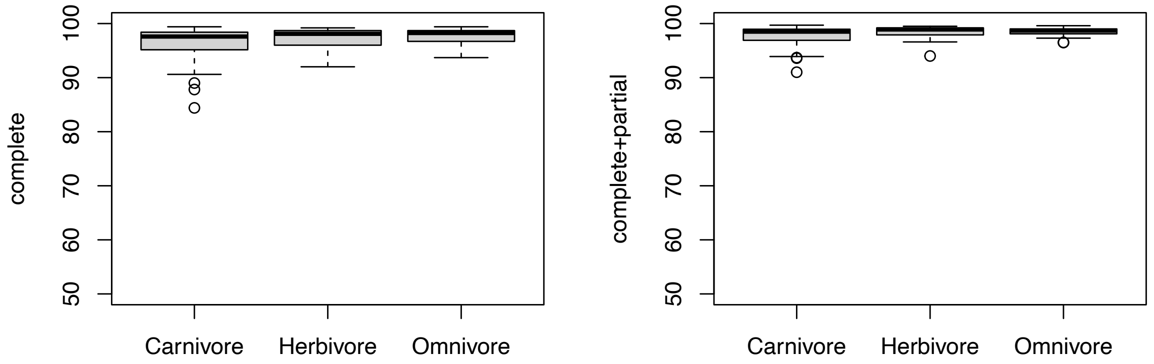

Fig. S1
Boxplots of BUSCO scores (left, complete; right, complete+partial) in different diet categories.

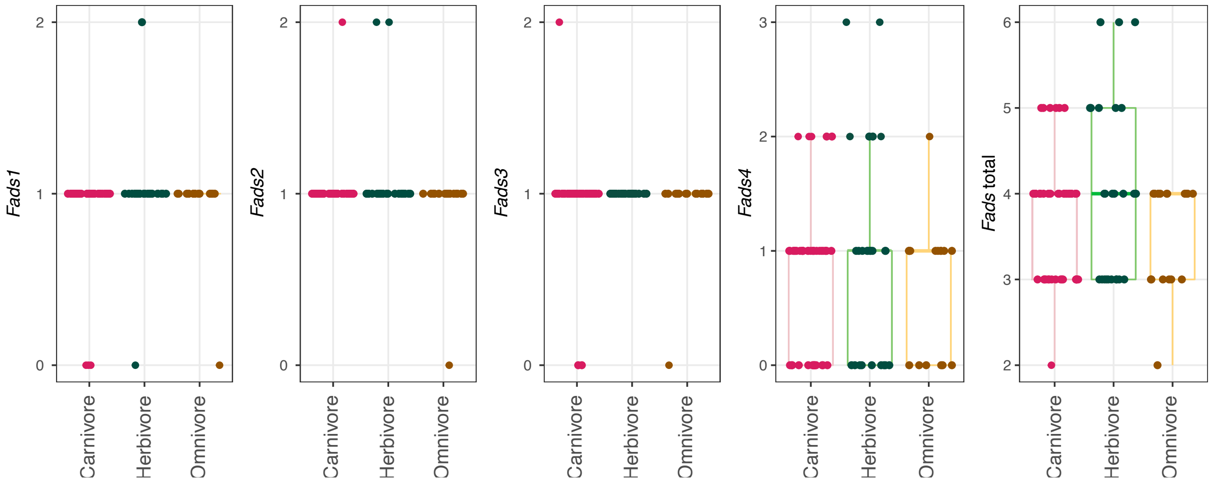

Fig. S2
Copy number differences of *Fads1-4* and total number of all *Fads* genes among different trophic categories. ORTHOSCOPE identifies sets of orthologs from publicly available databases of protein-coding genes constructed from whole genome sequence data. ORTHOSCOPE collects the longest transcript from a single locus when multiple transcripts, such as alternative splice variants, exist. In order to delineate each mammalian *Fads* gene lineage, gene models of non-mammalian vertebrates (*Gasterosteus aculeatus*, *Oryzias latipes*, *Xenopus laevis*, *Anolis carolinensis*, *Python bivittatus*, *Pelodiscus sinensis*, *Crocodylus porosus*, *Gallus gallus*, and *Taeniopygia guttata*) were also used in addition to 45 mammals. The following parameters were used: Mode = Search/Rearrangement; Focal group = Mammalia; E-value threshold = 1e-3; Number of hits to report per genome = 3; Dataset = DNA (Exclude 3rd). ORTHOSCOPE automatically generates a neighbor-joining tree of collected candidate orthologs and closely related genes. Using these trees, we selected *Fads* 1–4 orthologs based on the tree topology and counted the number of genes for each of 45 mammalian species. None of them were significant by Kruskall-Wallis test: chi-squared = 1.0854, p = 0.5812 for *Fads1*; chi-squared = 4.161, p = 0.1249 for *Fads2*; chi-squared = 0.76212, p = 0.6831 for *Fads3*; chi-squared = 0.5413, p = 0.7629 for *Fads4*; chi-squared = 1.9758, p = 0.3724 for total *Fads*.

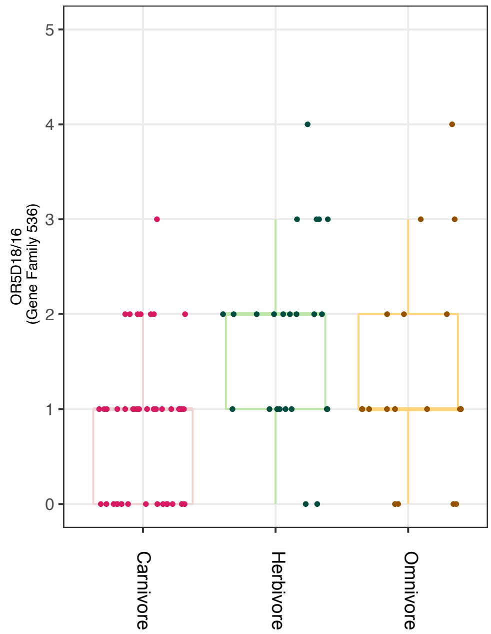

Fig. S3
Plot of gene copy number differences of gene family 536 (OR5D16/18) after removing one outlier species (*Loxodonta africana*).
